# Rhodopsin: A Potential Biomarker for Neurodegenerative Diseases

**DOI:** 10.3389/fnins.2020.00326

**Published:** 2020-04-15

**Authors:** Cameron Lenahan, Rajvee Sanghavi, Lei Huang, John H. Zhang

**Affiliations:** ^1^Burrell College of Osteopathic Medicine, Las Cruces, NM, United States; ^2^Center for Neuroscience Research, Loma Linda University School of Medicine, Loma Linda, CA, United States; ^3^Department of Neurosurgery, Loma Linda University School of Medicine, Loma Linda, CA, United States; ^4^Department of Physiology and Pharmacology, Loma Linda University School of Medicine, Loma Linda, CA, United States; ^5^Department of Anesthesiology, Loma Linda University School of Medicine, Loma Linda, CA, United States

**Keywords:** rhodopsin, amyotrophic lateral sclerosis, Parkinson’s disease, Alzheimer’s disease, Huntington’s disease, confocal scanning laser ophthalmoscopy

## Abstract

Retinal alterations have recently been associated with numerous neurodegenerative diseases. Rhodopsin is a G-protein coupled receptor found in the rod cells of the retina. As a biomarker associated with retinal thinning and degeneration, it bears potential in the early detection and monitoring of several neurodegenerative diseases. In this review article, we summarize the findings of correlations between rhodopsin and several neurodegenerative disorders as well as the potential of a novel technique, cSLO, in the quantification of rhodopsin.

## Introduction

Neurodegenerative disease is a major problem faced by an aging population around the world (Prince et al., 2013). As the population of elderly patients aged 65 and older will likely double between 2000 and 2030, the prevalence of age-related diseases is expected to substantially increase (Jedrziewski et al., 2007). These disorders are characterized by genetic mutations, which in turn upset protein homeostasis, leading to a variety of clinical manifestations. Current brain imaging techniques, such as magnetic resonance imaging, enable detection of cerebral atrophy or measure metabolic changes, which help to diagnose neurodegenerative pathologies (Schaller, 2008). However, certain pathologies seen on imaging are often detected only after the disease has progressed (Schaller, 2008). Moreover, current imaging, such as PET scans, would be difficult to implement for population-wide screening of preclinical signs due to the expense or necessity of radioactive isotopes (Colligris et al., 2018). While the molecular mechanisms of distinct neurodegenerative diseases may vary, there are also shared characteristics, such as neurite retraction and neuronal death (Bredesen, 2009). Given the nature of neuronal death, treatment for late stages of neurodegenerative diseases, such as Alzheimer’s, have been ineffective, therefore requiring early diagnosis (Sheinerman and Umansky, 2013). Should screening become possible at earlier stages, clinicians can better intervene in the progression of these diseases. Retinal alterations have recently been associated with numerous neurodegenerative diseases (Helmer et al., 2013; Sivak, 2013; Tanito and Ohira, 2013; Hubers et al., 2016; Mukherjee et al., 2017; Sudharsan et al., 2017; Ahn et al., 2018; Satue et al., 2018; Chiquita et al., 2019; Cipollini et al., 2019; den Haan et al., 2019; Dhalla et al., 2019). Rhodopsin, a G-protein coupled receptor in the rod cells of the retina is a biomarker associated with retinal thinning and degeneration (Cideciyan et al., 2005; Xiong and Bellen, 2013), suggesting its potential in the early detection and progression monitoring of neurodegenerative diseases. In this review, we summarize physiological function of rhodopsin, research findings of correlations between rhodopsin with several neurodegenerative disorders, and a novel technique, cSLO, in rhodopsin quantification.

## Rhodopsin

**FIGURE 1:**
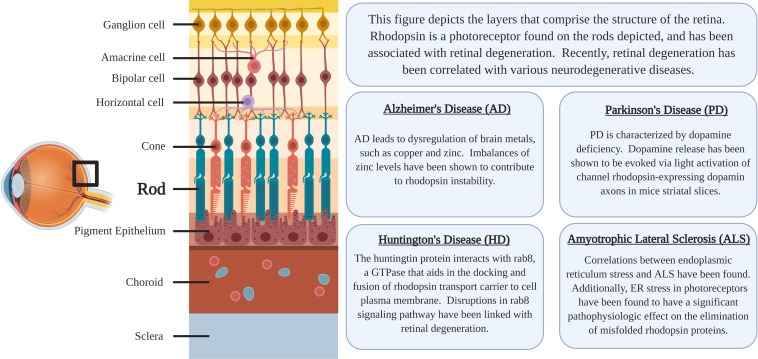
The location of rhodopsin-expressing Rod cells within the retina and a summary of the associations regarding neurodegenerative diseases and rhodopsin pathology.

**FIGURE 2:**
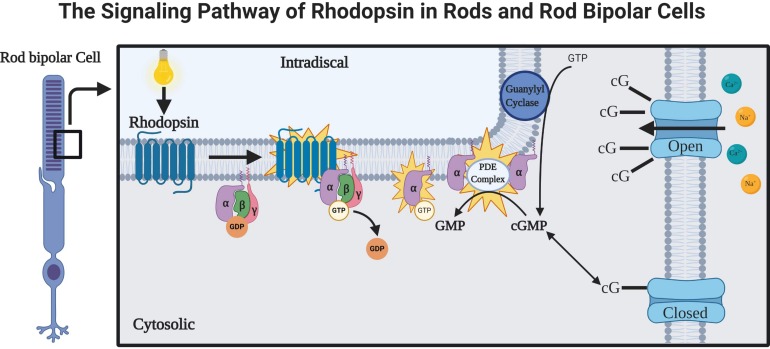
The rhodopsin-mediated signal transduction cascade in rods and rod bipolar cells (adapted from Pahlberg and Sampath, 2011). As light is absorbed by rhodopsin, a G-protein-coupled receptor (GPCR), the activations of phototransduction in the photoreceptors initiate an exchange of GTP and GDP. The cGMP is hydrolyzed by a cGMP phosphodiesterase complex (PDE complex). This reduction in the concentration of cGMP will close the cGMP-gated channels, which function to depolarize the membrane potential of rod bipolar cells via influx of Na^+^ and Ca^2 +^ (Pahlberg and Sampath, 2011).

Rhodopsin is a G-protein coupled receptor, and is the most abundant protein in the rod cells found in the retina (Figure 1). It functions as the primary photoreceptor molecule of vision, and contains two parts: an opsin molecule linked to a chromophore, 11-*cis*-retinal (Athanasiou et al., 2018). The opsin molecule is comprised of 348 amino acids, and has seven transmembrane domains (Athanasiou et al., 2018). Rhodopsin is synthesized in the rough endoplasmic reticulum of the inner segments of photoreceptors and subsequently undergoes posttranslational modifications in the Golgi before becoming functional (Murray et al., 2009). In Figure 2, an image adapted from an article by Pahlberg and Sampath, 2011 depicts the signaling pathway of rhodopsin. When light activates rhodopsin, phototransduction occurs, initiating the exchange of GDP for GTP on the G-protein, transducing (G_t_α), consequently increasing cGMP (or cG) hydrolysis through the PDE complex (Pahlberg and Sampath, 2011). If the cGMP concentration decreases, the cGMP-gated channels will close, preventing depolarization induced by the influx of Na^+^ and Ca^2+^. Therefore, activation of rhodopsin from photons of light is consequently followed by a small, graded hyperpolarization in membrane potential (Pahlberg and Sampath, 2011).

Rhodopsin’s function as a photoreceptor also allows its participation in circadian rhythm. Degeneration of these photoreceptors may manifest as a gradual thinning of the outer nuclear layer, a reduction of electroretinogram amplitudes, and vision loss (Xiao et al., 2019). A recent study by Ni et al. (2017) found that rhodopsin receptors in drosophila functions as a circadian pacemaker in neurons. Other studies have found that disruptions of normal circadian rhythms can have significant effects on health, and potential mechanisms have been suggested to link circadian dysfunction and neurodegenerative diseases (Musiek and Holtzman, 2016).

Microbial rhodopsins have been discovered in various species throughout the animal kingdom, and have aided in our understanding of neuronal functions (Duebel et al., 2015). In these microbes, they play an important role in photosynthesis and phototaxis. Examples of microbial rhodopsins include Channelrhodopsin, bacteriorhodopsin, and archaerhodopsin (Zhang et al., 2011). Channelrhodopsins were first isolated from the alga *Chlamydomonas reinhardtii* by Nagel et al. (2002). These channelrhodopsins are light-gated cation channels that can induce neuronal depolarization in response to stimulation from light (Daadi et al., 2016). Channelrhodopsin and its variants have since been used in research to manipulate cell membrane potentials using light energy (Lin, 2011). The opsin component of these proteins have rapid kinetics and are structurally simplistic, allowing neuronal expression using optogenetics (Zhang et al., 2011). Therefore, rhodopsin has emerged as a biomarker, which may serve as the link between retinal thinning and neuronal pathology seen in neurodegenerative diseases.

## Rhodopsin and Retinal Degeneration

In recent years, retinal thinning has become heavily associated with neurodegenerative diseases of the brain (Helmer et al., 2013; Sivak, 2013). There have been correlations established between retinal thinning and Alzheimer’s disease (Chiquita et al., 2019; Cipollini et al., 2019; den Haan et al., 2019), Parkinson’s disease (Ahn et al., 2018; Satue et al., 2018), Huntington’s disease (Dhalla et al., 2019), Amyotrophic lateral sclerosis (Hubers et al., 2016; Mukherjee et al., 2017), and a case has been reported of reduced retinal thickness after an occipital lobe infarction (Tanito and Ohira, 2013). Given this, it is important that we consider exploring biomarkers of the retina as possible routes of monitoring and diagnosing these debilitating neurodegenerative diseases. In recent years, more studies have explored the relationship between the two. Sudharsan et al. (2017) found that light-induced damage of the photoreceptors consequently led to a thinning of the outer nuclear layer, induction of Müller cell gliosis, focal loss of retinal pigment epithelial cell integrity, and an increased expression of endothelin receptor B in Müller cells. The retinal pigment epithelial layer is a monolayer of pigmented cells, known to contribute significantly, functioning as both a barrier and as an immunosuppressant in the eye (Touhami et al., 2018). Unsurprisingly, the relationship between the retinal pigment epithelial cells and photoreceptor cells is vitally necessary for sight (Sparrow et al., 2010). As previously described, Xiao et al. (2019) revealed that rhodopsin knockout mice had a reduction of electroretinogram amplitudes, vision loss, and gradual thinning of the outer nuclear layer of the retina. Correlations have already been established between rhodopsin and the conditions that are associated with retinal thinning, such as retinitis pigmentosa and age-related macular degeneration (Liu et al., 2015). More specifically, while changes in the retina may be early indicators for neurodegeneration, changes in rhodopsin have been shown to contribute to the early events of retinal degeneration (Di Pierdomenico et al., 2017), lending credibility to its potential as an even earlier biomarker for diagnosis.

## Rhodopsin and Alzheimer’s Disease

Alzheimer’s disease (AD) is a degenerative disease involving cortical atrophy, neuritic plaques, and neurofibrillary tangles (Hardy and Allsop, 1991), and is the most common dementia among the aging population (Reitz et al., 2011). Approximately 30 million people suffer from Alzheimer’s disease, and this number is expected to triple over the next 20 years (Jahn, 2018). The pathophysiology of AD lies in the buildup of misfolded extracellular amyloid plaque and intracellular neurofibrillary tangles in the brain (Reitz et al., 2011). It is currently believed that toxic amyloid plaques serve as the earliest manifestation of the disease (Albert et al., 2011; McKhann et al., 2011; Sperling et al., 2011; Weller and Budson, 2018). More recently, Manifestations of AD have been discovered in many different parts of the eye, such as the pupil, lens, choroid, and optic nerve, although this research has been minimally used in the clinic (Javaid et al., 2016). A study conducted by Yoon et al. (2019) found microvascular retinal changes both in patients with the disease, as well as patients with mild cognitive impairment, a notable precursor to Alzheimer’s disease. Amyloid deposits have also been found in the retina (Koronyo-Hamaoui et al., 2011). One study has found a correlation between the thinning of the retinal nerve fiber layer (RNFL) and retinal ganglion cell degeneration leading to the progression of Alzheimer’s disease (Javaid et al., 2016). For example, Mutlu et al. conducted a cohort study, and found that the thinner RNFL was associated with a greater risk for developing AD, independent of cardiovascular risk factors. Furthermore, studies on post-mortem specimens revealed that patients with AD had a substantial loss of retinal ganglion cells and thinner RNFL compared to those who did not have AD (Mutlu et al., 2018). These results suggest that retinal thinning occurs in the earlier stages of the disease, potentially enabling earlier diagnosis.

One study proposes a mechanism where rhodopsin misfolding ultimately contributes to retinal degeneration and the visual changes that are seen in both retinitis pigmentosa and Alzheimer’s disease (Stojanovic et al., 2004). Surprisingly, recent research has suggested that the dysregulation of brain metals, such as copper and zinc, occurs in AD (Sensi et al., 2018). In addition, zinc supplementation positively affects neurotrophic signaling and activation of the brain-derived neurotrophic factor (BDNF)-TrkB axis (Hwang et al., 2005; Sensi et al., 2009; Corona et al., 2010). Conversely, zinc deficiency contributes to retinal neurodegeneration and night blindness (Ugarte and Osborne, 2001). Stojanovic et al. (2004) found that mutations leading to these visual changes involve a high-affinity transmembrane site of rhodopsin that coordinates zinc, showing that zinc plays an important role in rhodopsin stability. However, at excess concentrations, zinc actually reduces the thermal stability ability of rhodopsin to link with 11-*cis-*retinal (del Valle et al., 2003). Too much zinc also promotes the aggregation of amyloid and prions in addition to rhodopsin. Therefore, a delicate balance of zinc is crucial in maintaining adequate rhodopsin stability (Stojanovic et al., 2004).

A study by Reddy et al. (2017) described the effects of Alzheimer’s on the retina, and found that the architectural disruption of the retina was associated by a loss of rhodopsin, increased gliosis, and retinal thinning.

## Rhodopsin and Parkinson’s Disease

Dopamine imbalance is involved in many neurodegenerative disorders, such as schizophrenia and Parkinson’s disease (PD) (Birtwistle and Baldwin, 1998). PD is the second most common neurodegenerative disorder in the United States (Kowal et al., 2013), and is caused by a loss of dopaminergic neurons of the substantia nigra pars compacta (Miller and O’Callaghan, 2015). The symptoms of PD usually appear after approximately 80% of these neurons have been lost (Altintas et al., 2008). Histologic findings reveal accumulation of alpha-synuclein bound to ubiquitin, forming Lewy Body inclusions, which are involved in disease pathology (Schulz, 2007). It is characterized by tremor, cogwheel rigidity, akinesia/bradykinesia, postural instability, and shuffling gait (Miller and O’Callaghan, 2015). In PD, changes in visual function have been reported, such as decline in visual acuity, contrast sensitivity, color vision, motion perception, and bioelectrical activity (Archibald et al., 2009).

Ahn et al. (2018) studied retinas in individuals who were recently diagnosed with PD using optical coherence tomography (OCT). They propose that retinal thinning in the macular area occurs early during the course of PD and corresponds to disease severity. These retinal changes were observed in patients who had been diagnosed with PD 2 years prior, but had not started a medication regimen (Ahn et al., 2018). Their research reveals that retinal thinning may be connected with the loss of dopaminergic neurons. Retinal thinning in the inner plexiform and ganglion cell layers was discovered in these patients. However, the degree of thinning also corresponded to the subjects’ scores on the Hoehn and Yahr scale, a measure of PD progression (Goetz et al., 2004). Retinal thinning in this area also correlated with decreased dopamine transporter activity in the left substantia nigra (Ahn et al., 2018). Most interesting, a recent study indicates that dopamine release was evoked via light activation of channel rhodopsin-expressing dopamine axons in mouse striatal slices (Brimblecombe et al., 2015). Therefore, it is reasonable to further explore the impact that rhodopsin concentrations may have in diagnosing this devastating disease, and further supports the hypothesis that rhodopsin may play a pivotal role in early diagnosis.

## Rhodopsin and Huntington’s Disease

Huntington’s disease (HD) is an autosomal dominant disorder caused by trinucleotide repeat expansion (CAG) in the huntingtin gene on chromosome 4 (Jordan and Wright, 2010). This leads to atrophy of the caudate and putamen, resulting in increased levels of dopamine and decreased levels of GABA and acetylcholine in the brain. It is characterized by chorea, athetosis, depression, and mental decline in later stages.

OCT imaging was also used to measure the thickness of the retinal layers using foveal scans in patients with Huntington’s disease. Compared to healthy control patients, there was reduced thickness in the macular RNFL and ganglion cell layer in those with Huntington’s disease, which also corresponded to disease progression markers (Gulmez Sevim et al., 2019). Another study revealed significant abnormalities in color vision in HD patients who had reduced thickness of the temporal portion of the RNFL (Kersten et al., 2015). Moreover, Knapp et al. (2018) followed a presymptomatic patient that had tested positive for the gene responsible for HD, and found rod dysfunction within both eyes. There are numerous proteins involved with rhodopsin trafficking, such as rab8. Disruptions in this rab8 signaling pathway have also been linked to retinal degeneration (Moritz et al., 2001). Rab8 is a GTPase that aids in the docking and fusion of rhodopsin transport carrier to the cell plasma membrane. They concluded that the huntingtin protein interacts with rab8, and is involved in linking cellular organelles to cytoskeletal components (Deretic, 2006), demonstrating a parallel mechanism between breakdown of cell trafficking in retinal degeneration and HD.

The aggregation of P23H, a mutated rhodopsin found in retinal cells and seen in retinitis pigmentosa, leads to the impairment of the ubiquitin proteasome system, an important protein degradation system (Bence et al., 2001). A mutated form of the huntingtin protein aggregates and impairs protein degradation in the same manner, according to Illing et al. (2002). Therefore, the aggregation of protein in Huntington’s disease introduces the possibility that retinal degeneration may share a common mechanism with neurodegeneration of the retina.

While retinal thickness has been associated with HD, there have not been any confirmed studies regarding rhodopsin and HD. However, it is likely that HD would follow the same pathological patterns as the other neurodegenerative diseases. Future studies are necessary to determine any possible correlation with rhodopsin and Huntington’s, and whether it has potential as an early diagnostic biomarker.

## Rhodopsin and Amyotrophic Lateral Sclerosis

Amyotrophic lateral sclerosis (ALS) is the most common motor neuron disease in adults (Talbott et al., 2016). It is an unrelenting disease resulting in progressive paralysis, worsening until death is caused by respiratory failure (Al-Chalabi and Hardiman, 2013). The disease is distinct, in that it is characterized by upper and lower motor neuron involvement (Al-Chalabi and Hardiman, 2013). The most common gene mutations for ALS have been identified as C9orf72, SOD1, TARDBP, and FUS (Zou et al., 2017).

Several studies have found a positive correlation between retinal thinning and ALS patients (Hubers et al., 2016; Mukherjee et al., 2017; Rojas et al., 2019). However, loose connections may be made regarding ALS and rhodopsin, as one study has found a correlation between ER stress and ALS pathogenesis (Webster et al., 2017). Subsequently, ER stress in photoreceptors also has a significant pathophysiological effect on the elimination of misfolded rhodopsin proteins (Chiang et al., 2015). There is also a connection regarding the unfolded protein response inositol-requiring enzyme 1 (IRE1) signaling pathway. This pathway is strongly activated in misfolded rhodopsin-expressing photoreceptors, and significantly upregulated P23H rhodopsin degradation (Chiang et al., 2015). Similarly, ALS mice had increased IRE1 prior to symptom onset (Jaronen et al., 2014). While the evidence is not conclusive for a connection, it does provide sufficient evidence to warrant future inquiry.

## Rhodopsin and Stroke

Hypertension and cerebrovascular changes, including stroke, increase the risk of cognitive impairment (Reitz et al., 2011). Ischemia occurs when blood supply has been restricted to an area due to blockage of blood vessels, resulting in dysregulation and cell death (Joachim et al., 2017). Likewise, retinal ischemia can result from a lack of perfusion due to a blockage of capillaries. Inflammation follows several hours after onset of ischemia, consequently leading to apoptosis (Osborne et al., 2004). Joachim et al. describes an animal model of retinal ischemia-ischemia reperfusion (I/R), in which pressure in the eye is temporarily increased through the infusion of liquid into the anterior chamber, leading to the compression of vasculature supplying the optic disc and loss of neuronal cells in the retina. They found that the thickness of the inner retinal layers is reduced first, and prolonged ischemia results in insult to the outer retinal layers, including the photoreceptors (Joachim et al., 2017).

Researchers propose that decreased contents of photoreceptor proteins represent an early stage of retinal degeneration. Retinal vein occlusion (RVO), the second most common retinal vascular disease after diabetic retinopathy, results from the compression of the retinal vein as a result of atherosclerosis or increased blood viscosity (Khayat et al., 2018). Coincidentally, individuals with RVO are also at a significantly greater risk of developing ischemic and hemorrhagic strokes (Chen et al., 2018). In one study, RVO was induced in pigs, which led to a reduction in proteins involved in vision, such as rhodopsin (Cehofski et al., 2018). This compromise in rhodopsin function suggests that neurotransmission is sensitive to ischemic insult. However, it is lacking specific research exploring the possible relationship between stroke and rhodopsin.

## Rhodopsin Quantification and Scanning Laser Ophthalmoscopy

Scientists at Florida International University have developed a novel technique, known as nano-second pulsed scanning laser ophthalmoscopy (SLO), which was established as an effective method of imaging rhodopsin (Liu et al., 2015). Other novel methods of rhodopsin imaging have also been proposed, such as the confocal laser ophthalmoscope (cSLO), which creates high-resolution spatial mapping of rhodopsin and retinal pigment epithelium distribution (Ehler et al., 2015). This proposed technique, using cSLO, is a novel, non-invasive *in vivo* method, and functions by analyzing the brightening of detected lipofuscin autofluorescence within small pixel clusters, creating images of ∼50-μm resolution (Ehler et al., 2015). Furthermore, to emphasize the clinical relevance of this technique, it was designed to be used with widely available clinical imaging devices (Ehler et al., 2015). It has recently been proposed that OCT may be used in various neurodegenerative diseases, due to its capability in measuring volumetric changes of the retina (Doustar et al., 2017). However, given the nature of neurodegenerative diseases, it is paramount that the diagnosis is established at the earliest possible time-point to provide the best prognosis to the patient. Therefore, cSLO warrants further research to determine the efficacy and correlation between rhodopsin concentrations and disease progression of neurodegenerative disorders.

## Conclusion and Future Direction

There are several limitations that should be addressed. For example, the unfolded protein response not only occurs neurodegenerative disorders, but is also activated as a part of the normal aging process, which is marked by an increase in oxidative stress and the pro-inflammatory state (Lenox et al., 2015). Jackson et al. hypothesized that aging results in disturbance of the visual cycle responsible for rhodopsin regeneration. Their investigation revealed that the transition point in the dark, at which the rod system is the primary contributor to vision, was delayed by nearly 2.5 min in elderly population compared to young adults, resulting in a slower rate of dark adaptation kinetics seen in older adults (Jackson et al., 1999). This presents limitations in using rhodopsin as a biomarker, as more research is needed to discern reduction in rhodopsin as a biomarker for age-related changes versus disease pathology.

Currently, there are also limitations in specificity regarding rhodopsin and its potential use in diagnosis. Because of the lack of rhodopsin quantification in the studies described above, it would be difficult to identify one neurodegenerative disorder specifically, but may have potential, if used with other clinical presentations as a quick, non-invasive confirmatory test. Future studies should investigate and determine whether the rates of rhodopsin degeneration differ among neurodegenerative diseases.

With the substantial evidence suggesting a correlation between retinal thinning, rhodopsin levels, and progression of neurodegenerative diseases, there is a clearly defined need to explore whether this biomarker is more beneficial in the early detection and monitoring of these debilitating conditions. While there have not yet been any studies that attempt to correlate rhodopsin with Huntington’s, or ALS, there are various genes and signaling pathways that suggest a possible relationship. Furthermore, rhodopsin has been associated with retinal degeneration, a recently discovered indicator of neurodegenerative diseases.

For each study that we found including rhodopsin with retinal thinning and degradation, it appears that the rhodopsin levels simultaneously decreased. Given that changes in rhodopsin levels are known to precede alterations of the retina, it is logical to suggest that research is warranted to determine whether the absence of rhodopsin is significant in diagnosing these neurodegenerative disorders. With the development of novel techniques, such as cSLO, which allows the quantification of rhodopsin, efforts should be made to determine whether rhodopsin might have an increased sensitivity for the diagnosis and monitoring of neurodegenerative diseases. Measuring rhodopsin levels will likely be a beneficial complementary biomarker used in conjunction with assessing retinal thickness via OCT.

## Author Contributions

CL and RS drafted the manuscript. LH assisted with revisions. CL, RS, LH, and JZ conceived of this study. All authors read and approved the final manuscript.

## Conflict of Interest

The authors declare that the research was conducted in the absence of any commercial or financial relationships that could be construed as a potential conflict of interest.

## Abbreviations

AD: Alzheimer’s disease
ALS: amyotrophic lateral sclerosis
cSLO: confocal scanning laser ophthalmoscopy
HD: Huntington’s disease
OCT: optical coherence tomography
PD: Parkinson’s disease
RNFL: retinal nerve fiber layer
SLO: scanning laser ophthalmoscopy.
